# Design and evaluation of the I-SCAN faculty POCUS program

**DOI:** 10.1186/s12909-020-02453-2

**Published:** 2021-01-06

**Authors:** Michael Janjigian, Anne Dembitzer, Caroline Srisarajivakul-Klein, Khemraj Hardower, Deborah Cooke, Sondra Zabar, Harald Sauthoff

**Affiliations:** 1 Department of Medicine, New York University Grossman School of Medicine, NYC Health & Hospitals/Bellevue, New York, USA; 2 Department of Medicine, New York University Grossman School of Medicine, VA NY Harbor Healthcare System, New York, USA; 3 Department of Medicine, New York University Grossman School of Medicine, NYU Langone Health, New York, USA

**Keywords:** Point-of-care ultrasound, Medical education, Program assessment

## Abstract

**Background:**

Point-of-care ultrasound (POCUS) is becoming widely adopted with increasing accessibility of courses. Little is known about the optimal design of the introductory course or longitudinal training programs targeting hospitalists that are critical to success.

**Methods:**

Hospitalists at four academic sites participated in a two-day introductory course and a longitudinal phase comprising clinical POCUS practice, clip uploading with online feedback, hands-on teaching, and monthly ultrasound conferences. Assessments were performed immediately before and after the two-day course and after 1 year.

**Results:**

Knowledge increased from baseline to post two-day course (median score 58 and 85%, respectively, *p* < 0.001) and decreased slightly at 1 year (median score 81%, *p* = 0.012). After the two-day introductory course, the median score for hands-on image acquisition skills, the principal metric of participant success, was 75%. After 1 year, scores were similar (median score 74%). Confidence increased from baseline to post two-day course (1.5 to 3.1 on a 4 point Likert scale from Not at all confident (1) to Very confident (4), *p* < 0.001), and remained unchanged after 1 year (2.73). Course elements correlating with a passing score on the final hands-on test included number of clip uploads (r = 0.85, *p*,0.001), attendance at hands-on sessions (r = 0.7, *p* = 0.001), and attendance at monthly conferences (r = 0.50, *p* = 0.03).

**Conclusions:**

The I-ScaN POCUS training program increased hospitalist knowledge, skill and confidence with maintained skill and confidence after 1 year. Uploading clips and attending hands-on teaching sessions were most correlative with participant success.

## Background

Point-of-care ultrasound (POCUS) is becoming widely adopted across the field of hospital medicine after becoming well established in fields with competency overlap such as Emergency Medicine and Pulmonary and Critical Care Medicine [1, 2]. The reduction in procedural complications, improvement in diagnostic accuracy, and an increase in provider and patient satisfaction has pushed POCUS expansion [3–7]. In addition, the availability of less expensive ultrasound devices and the increasing accessibility of training programs have facilitated the growth of POCUS within general medicine, both in the inpatient and ambulatory settings [2, 8–10].

A 2013 survey of internal medicine program directors found that POCUS was considered valuable for diagnostic and procedural use and many residency programs had either adopted formal curricula or had planned to [11]. A similar study of UME leadership in 2014 reported general consensus that POCUS is an important skill worth teaching in medical school [12]. A recent position statement from the Alliance of Academic Internal Medicine recommended the integration of POCUS across the longitudinal training environment for UME, GME and CME in internal medicine [1].

However, the training of faculty has emerged as a key barrier to adoption into hospitalist groups [2, 13–15]. Published reports of comprehensive faculty POCUS training programs demonstrate immediate short-term gains as would be expected, however, there is limited data on durability of these gains [14–17]. The CHAMP program reported that skills learned in the introductory course tended to wane over time, with participation in monthly scanning sessions/and or portfolio completion being associated with skill retention [14]. The recent position statement issued by the Society of Hospital Medicine (SHM) advocates for attendance at a local or national hands-on training program, which should be followed by a longitudinal study phase with hands-on instruction [2]. Educational outcome data to guide faculty POCUS program development are lacking.

We sought to address concerns of educational durability as we developed and implemented the Integrated Sonography Course at NYU (I-ScaN), a longitudinal POCUS training program for hospitalist faculty. We describe the design, implementation and 1-year outcomes of an observational cohort study of the I-ScaN program.

## Methods

### Setting and participants

The NYU Department of Medicine encompasses four teaching hospitals, NYU Langone Health (Tisch/Kimmel and Brooklyn campuses), Health + Hospitals/Bellevue, and the VA New York Harbor Health Care System/Manhattan. Each campus has a unique structure of teaching and non-teaching services and populations served. All physicians who were identified by their directors as core hospitalists, generally defined as those faculty who spend the majority of their clinical time on the inpatient teaching service, were invited to participate. Fifty percent (23/46) of the physicians who were asked accepted the invitation to participate.

### Program description

I-ScaN is a faculty development program with the goal of developing a cohort of physicians proficient in POCUS who will foster a culture of ultrasound use across the institution through routine clinical practice of POCUS and who will have the ability to supervise and educate the residents and students in turn. The program consists of four elements: 1. Introductory course including didactics, image interpretation and hands-on instruction; 2. Supervised practice with patients; 3. Competency assessment; 4. Skill maintenance and quality assurance [18]. I-ScaN adheres to ultrasound applications and a training structure advocated by SHM [2].

I-ScaN began with a period of self-study upon program registration, where participants were referred to relevant chapters from a POCUS textbook, [18] online videos and selected articles to review. The self-study period lasting approximately 1 month was followed by an intensive two-day course in April of 2018. The course consisted of didactic lectures reviewing theoretical concepts, interactive image-based sessions of interpretation and clinical integration, and hands-on training on human models. To shorten the three-day American College of Chest Physicians Critical Care Ultrasound course upon which the two-day I-ScaN course was modeled,[16] we excluded elements such as the pre-assessment hands-on testing, critical care application lectures, and the credentialing and economics lecture, while truncating slightly the hands-on sessions. Systems covered in the course included cardiac ultrasound (five standard views), lungs/pleura, abdomen (kidneys, bladder and aorta), and leg vasculature. Participants reviewed both normal and a range of abnormal scans during the Image interpretation sessions.

Following the two-day course, participants began the longitudinal portion of the program. An ultra-portable ultrasound device with the ability to upload ultrasound clips to a HIPAA-compliant website was provided to the faculty on each inpatient teaching service. Participants also had access to traditional cart-based ultrasound machines at each hospital. Participants were advised to scan patients daily when on service and upload all clips, anonymized to avoid sharing protected health information, to secure servers allowing comments and expert review. For each uploaded clip, participants were asked to provide the anatomic site and a clinical interpretation. The clips were reviewed by an expert (HS) who provided online feedback on both of these domains. Participants used these clips to create a personal portfolio in accordance with national standards set by SHM [19].

Hands-on teaching sessions were led by local experts, primarily Pulmonary and Critical Care Medicine faculty, with a 1: 3 teacher to participant ratio. Sessions typically lasted one hour with the entire time spent at the bedside scanning a patient known to one of the participants. Each of the four hospitals arranged these sessions according to participant and teacher availability with the aim for weekly supervised scanning.

Ultrasound conference, led by the course director (HS), was held monthly with remote viewing capability. Conferences allowed for a participant to present saved clips and a brief lecture to their peer group. Presenters and topics were rotated each month.

### Assessments

**Fig. 1:**
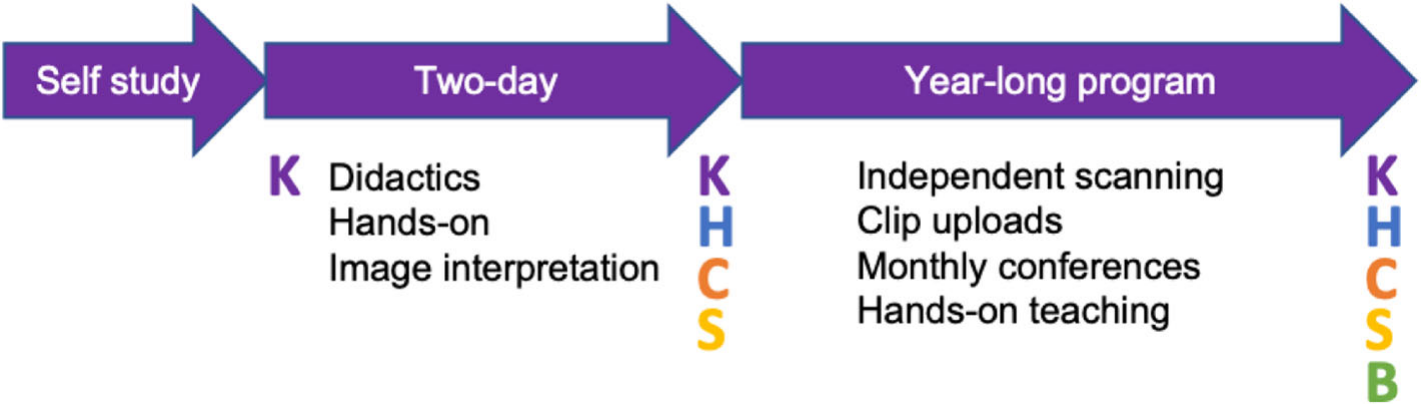
Timeline of program assessments. Abbreviations: K, Knowledge test; H, Hands-on test; C, Confidence survey; S, Satisfaction survey; B, Barriers survey

We sought to assess participant outcomes at multiple levels of Miller’s Pyramid: basic ultrasound knowledge, application of this knowledge to patient care, demonstration of the technical skills of image acquisition, and integration of these new skills into everyday situations [20]. The timeline of program assessments is outlined in the Fig. 1.

Attendance was recorded for monthly conferences, including those who joined remotely, and at hands-on teaching sessions at each site. Total number of uploaded clips was recorded for each participant.

We assessed participants’ knowledge of basic ultrasound knowledge, image interpretation, and clinical integration using a novel 20-item online test developed for this program. The same test was administered prior to beginning the program, at the conclusion of the two-day course and again after 1 year. The test was given three times to first measure the impact of the two-day course and then to assess for durability or decay of knowledge over the year.

We evaluated participants’ POCUS skills using a hands-on test on human models that was proctored by faculty from the Pulmonary and Critical Care Medicine division. Skills were evaluated by the proctors, using a checklist adapted from the CHEST Certificate of Completion program [21]. The hands-on assessment was rated using a three point scale of poorly done, partially done, or well done. All proctors received an orientation on the assessment tool by the course director. Scores were calculated based on the percentage of items receiving the well done designation.

We assessed participant confidence in image acquisition and clinical integration using a retrospective pre- post- survey at the completion of the two-day course and again after 1 year (Appendix 1). Responses were rated based on a 4-point Likert scale from Not at all confident (1) to Very confident (4).

We also examined participants’ barriers to using POCUS. The barrier survey was developed from pilot interviews to address reasons participants may not adopt various aspects of POCUS (i.e. clinical use or clip uploading (Appendix 2)).

Participants completed a questionnaire regarding overall satisfaction with the program and individual elements.

Successful completion of the I-ScaN program required three components: 1) Achieving a score of ≥80% on the final knowledge test 2) Receiving a score of ≥80% on the hands-on test and 3) Submitting an image portfolio based on SHM guidance [19]. Passing scores were established at these values through author consensus of clinical relevance and review of published data [14].

### Statistical analysis

Pre- and post- two-day knowledge scores (Cronbach’s α = .68–.74) and confidence in ultrasound use (Cronbach’s α = .79–.84) were summarized. Scores from knowledge were reported as percentages and confidence scores reported on a 1–4-point scale. Differences between pre- and post-values for two-day course variables were assessed by using 2-sample paired Wilcoxon signed rank tests with a 95% confidence level. Data were reported as median and interquartile range for two-day and year end assessments (Table 1).

**Table 1:** Assessment scores before and after two-day course and after one-year

	Pre two-day Median (IQR)	Post two-day Median (IQR)	*p*
Knowledge (% correct)	58 (27)	85 (21)	*p* < 0.001
Confidence (1–4 Likert)	1.5 (0.55)	3.1 (0.62)	*p* < 0.001
	Post two-day Median (IQR)	One-year Median (IQR)	
Knowledge (% correct)	85 (21)	81 (24)	0.012
Confidence (1–4 Likert)	3.1 (0.62)	2.73 (0.85)	NS
Hands-on (% well done)	75 (28)	74 (30)	NS

*Abbreviations*: *IQR* interquartile range

Hands-on assessment was conducted at the end of two-days (Cronbach’s α = .84) and at the end of the program (Cronbach’s α = .93). Scores were summarized. At the end of the program, knowledge (Cronbach’s α = .74) and confidence (Cronbach’s α = .91) were reassessed and compared to post-two-days scores. Hands-on and knowledge scores were summarized and reported as percentages, while confidence scores are reported on a 1–4-point scale. Differences between two-days and end of program were assessed by using 2-sample paired Wilcoxon signed rank tests with a 95% confidence level. Data were reported as median and interquartile range for two-day and year end assessments (Table 1).

Spearman’s Correlation was performed on year-end hands-on assessment and knowledge, confidence, lectures, clips uploads, and hand-on lectures to understand the relationship between performance using POCUS and other reported metrics.

We considered a passing score on the final hands-on test to be the single best summative assessment to determine competency in POCUS skill as participants must demonstrate understanding of the ultrasound machine settings, demonstrate acquisition skills of each view, and identify key structures in each view.

The I-ScaN program qualified as a quality improvement project by the NYU Grossman School of Medicine’s Institutional Review Board criteria using a self-certification process to ensure the data were not collected for research purposes. The primary goal of the project was to assess and improve educational performance of the I-ScaN program.

## Results

Twenty-three hospitalists from across the 4 hospitals participated in the two-day introductory course. Sixteen of the participants (72%) reported prior ultrasound training, with a range of 2–80 h (median of 4 h); 3 reported more than 5 h of prior training. Three reported active clinical use of POCUS though none of them had more than 5 h of prior training. The group averaged 4.5 years of clinical practice (range = 1–13 years). Only the nineteen hospitalists who completed the assessments at the one-year mark are included in the subsequent analysis (3 left the institution, 1 voluntarily dropped out due to time commitments).

Approximately 1 year after the introductory course and at the time of the final hands-on test, participants had uploaded a total of 2787 clips (range 0–876, median 44), one had completed the image portfolio. At the time of writing four participants have satisfied all course elements.

Participant assessment scores from before and after the two-day course are presented in Table 1. Participant knowledge increased from before the two-day course to post-course (58 and 85% correct, respectively, *p* < 0.001). Knowledge scores fell slightly at 1 year (81% correct, *p* = 0.012). There was no change in POCUS skills as measured by the hands-on test from immediately after the two-day course to one-year (75 and 74%, respectively). Confidence ratings increased from 1.5 pre- to 3.1 post-course (out of possible 4, p < 0.001) and were 2.73 at one-year (NS for incremental change).

Correlation between course components and the hands-on test is presented in Table 2. Performance on the one-year knowledge test correlated highly with results of the one-year hands-on test (r = 0.78, *p* < 0.001), while correlation between the post-two-day knowledge and hands-on tests was only moderate (r = 0.53, *p* = 0.018). Participant confidence at 1 year positively correlated with hands on performance at 1 year (r = 0.55, *p* = 0.02), however confidence post two-day course did not correlate with hands on performance after the two-day course. There was a correlation between passing the one-year hands-on test with the number of clip uploads (r = 0.85, *p* < 0.001), attending hands-on teaching sessions (r = 0.7, *p* = 0.001), and with attendance at monthly conferences (r = 0.5, *p* = 0.03).

**Table 2:** Correlation between course components and hands-on test

	Post two-day hands-on		One-year hands-on	
	r	*p*	r	*p*
Post two-day knowledge	0.53	*p* = 0.018		
Post-two-day confidence		NS		
One-year knowledge			0.78	*p* < 0.001
Attendance at hands-on teaching sessions			0.70	*p* = 0.001
Attendance at monthly conference			0.50	*p* = 0.03
Clip uploading			0.85	*p* < 0.001
Confidence at one-year (*n* = 18)			0.55	*p* = 0.02

### Practice patterns

At one-year, 13/19 (68%) of participants strongly agreed or agreed that they felt confident using POCUS to make clinical decisions. Two (11%) reported using POCUS daily, 7 (37%) every 2–3 days, 1 (5%) weekly, and 9 (47%) “only with the right patient”. Lack of time during the workday was reported by 68% as the principal barrier to clinical use of POCUS. Scanning in front of house staff was reported infrequently as a deterrent (21% agreed or strongly agreed).

In responding to the prompt that “POCUS takes too much time”, participants perceived vascular studies to be the most time consuming and lung to be the least (37 and 11%, respectively). Planning to obtain a scheduled formal study was reported as a deterrent to performing POCUS for 21% of participants for cardiac views and 53% of participants for vascular views. Only 5–11% of participants reported not performing POCUS due to the physical exam alone being sufficient to make diagnoses across views.

Participants agreed or strongly agreed that the ultra-portable device, when compared to a traditional machine, poses less of a barrier regarding device access (16 and 53%), ability to carry/transport (5 and 79%), ease of uploading (26 and 74%), and labeling recorded images (53 and 63%).

### Program evaluation

At one-year, participants rated I-ScaN with an overall score of 4.6/5. Individual course elements were rated as follows: two-day course 3. 9, hands-on sessions 3. 9, and monthly conferences 3.4 (1 not at all useful to 4 very useful).

## Discussion

I-ScaN found that a two-day introductory course was effective at improving hands-on skill, knowledge and confidence, without a decline in hands-on skill following a longitudinal year-long curriculum. The critical portion of successful POCUS skill and knowledge acquisition lies in the longitudinal phase of the curriculum. Participants reported that lack of time was the principal barrier to adoption of POCUS.

Limited experience exists for the ideal POCUS training program for hospitalists. Maw and colleagues described a 10-week pilot program resulting in immediate improvements in ultrasound acquisition and interpretation though long-term outcomes were not studied [15]. The authors concluded that a longitudinal structured mentored training program would be required for participants to develop mastery. Cochard and others described a hospitalist POCUS training program where, of the 35 completing a survey out of 58 total who took the course, confidence waned at 6 months and 26% reported never using POCUS in their hospitalist practice [17]. Mathews, reporting on the CHAMP Ultrasound Program, found that of those faculty participating in a voluntary one-day refresher course following the initial three-day introductory course, skill retention was most effectively maintained by those who attended any monthly scanning session or who had started an image portfolio [14]. I-ScaN was designed to maximize the effectiveness of the longitudinal phase while shortening the introductory course to 2 days.

The sample sizes are too small to determine which program elements led to participant success, though similar to the results of the CHAMP program, we found that skill retention on the hands-on test correlated with attendance at monthly conferences, hands-on teaching sessions and image uploading. We believe that incorporation of scanning into clinical workflow immediately after the course along with routine direct supervision are the key elements that result in participant success in POCUS.

Participant confidence in using POCUS at 1 year correlated with their performance on the hands-on test at 1 year but not at the conclusion of the two-day course. These findings suggest that participants developed an awareness of their strengths and weaknesses over time as they progressed along the Dunning-Kruger curve [22]. Provider confidence may serve as a means of assessment when considering an individual’s scope of practice who has routinely integrated POCUS into clinical practice.

A strength of our study is the comprehensive data collection for all assessments and surveys of all 19 participants, save a single confidence assessment, representing a diverse faculty across 4 distinct practice settings, including those both highly and minimally engaged during the longitudinal portion of the curriculum. The studies reviewed above either report short-term results or only the highly-engaged fraction of the total cohort of long-term participants.

A recent survey of Internal Medicine faculty addressing barriers to POCUS adoption found that while the majority of respondents held a favorable view of POUCS, the most commonly cited barriers included the need for more training (79%), lack of a handheld ultrasound device (78%), lack of direct supervision (65%), and lack of an expert to review uploaded clips (53%) [23]. I-ScaN attempted to mitigate common barriers to ultrasound adoption through provision of ultra-portable devices to participants, by bringing teachers to the bedside of participants’ patients and providing expert review of all uploaded clips. Responses to our survey did not reveal a clear barrier apart from time, suggesting that I-ScaN was successful in addressing many common barriers. Participants found that the ultra-portable device was less of a barrier than a traditional machine for accessibility, portability, and ability to upload and label clips, supporting resource allocation in favor of these less expensive devices.

Though not specifically addressed on our survey, we believe the low completion rate of the image portfolio was due to the time required to practice, the knowledge gap in how to upload clips, and the lack of an incentive given that successful completion of I-ScaN was voluntary and only required for those who wanted to teach in the program or be allowed by local leadership to use POCUS in clinical care. We have subsequently included uploading of scans as a standard practice during hands-on teaching sessions to address this barrier. We anticipate that uploading of clips and portfolio completion by hospitalists will be commonplace once standards and expectations are set similarly to those found in fields such as Emergency Medicine.

Study limitations include a relatively small sample size encompassing a single cohort of participants, as is common in faculty development programs. The small sample size limits our ability to predict why some individual participants succeeded while others did not. Additionally, while we developed this program to foster a culture of POCUS by training core inpatient teaching faculty, we did not evaluate whether faculty did in-fact teach POCUS to others.

Despite ongoing encouragement and assessment of barriers throughout the longitudinal portion of the course, we found wide variance in faculty participation in program elements and practice patterns. Participants rated all elements of I-ScaN highly, reinforcing that it is the time spent between course elements that predicts durable gains. Getting participants to the bedside following an introductory course to practice on patients and upload clips for feedback will yield the greatest results.

## Conclusions

I-ScaN demonstrates that POCUS skill development learned after a two-day introductory course can be achieved through a longitudinal mentored program consisting of access to ultrasound devices, hands-on teaching sessions, and monthly case conferences. The lowered costs of ultra-portable ultrasound devices and availability of a growing population of instructors will facilitate the expected growth of hospitalists learning POCUS.

## Data Availability

The datasets during and/or analyzed during the current study available from the corresponding author on reasonable request.

## Appendix 1

**Table 3:** Retrospective Pre- Post Survey

Prior to taking this program	
How confident were you in your ability to: (all responses Not at all confident, Only a little confident, Somewhat confident, Very confident)	
acquire ultrasound (US) images of the heart?	
acquire US images of the lungs?	
acquire US images of abdominal organs?	
acquire US images of deep veins?	
interpret US images of the heart?	
interpret US images of the lungs?	
interpret US images of abdominal organs?	
interpret US images of deep veins?	
Prior to taking this program	
How likely were you to use ultrasound to? (all responses Not at all likely, Only a little likely, Somewhat likely, Very likely)	
evaluate dyspnea?	
evaluate abdominal pain?	
evaluate hypotension?	
evaluate volume status?	
evaluate for DVT?	
perform a paracentesis?	
insert a peripheral venous catheter?	
POCUS Questions:	
1) Have you received prior training in point of care ultrasound? (Yes/No)	
1a) If you answered yes to the question above, how many hours have you received in prior training? (free text)	
2) Do you use point of care ultrasound (Yes/No)	
Now that you have completed this program	
How confident were you in your ability to: (all responses Not at all confident, Only a little confident, Somewhat confident, Very confident)	
acquire ultrasound (US) images of the heart?	
acquire US images of the lungs?	
acquire US images of abdominal organs?	
acquire US images of deep veins?	
interpret US images of the heart?	
interpret US images of the lungs?	
interpret US images of abdominal organs?	
interpret US images of deep veins?	
Now that you have completed this program	
How likely were you to use ultrasound to? (all responses Not at all likely, Only a little likely, Somewhat likely, Very likely)	
evaluate dyspnea?	
evaluate abdominal pain?	
evaluate hypotension?	
evaluate volume status?	
evaluate for DVT?	
perform a paracentesis?	
insert a peripheral venous catheter?	

## Appendix 2

**Table 4:** I-ScaN One-Year Course Assessment

How useful did you find the following course elements? (all responses Not at all useful, Only a little useful, Somewhat useful, Very useful, N/A)	
Introductory 2-day course	
Monthly conferences	
Hands-on teaching sessions	
Brightspace site	
Do you feel you are competent to use POCUS to make clinical decisions (Strongly Agree, Agree, Disagree, Strongly Disagree)	
Overall how would you rate this course? (One of the worst, Below average, Average, Above average, One of the best)	
I feel comfortable with my POCUS skills (all responses Strongly Agree, Agree, Disagree, Strongly Disagree)	
Abdomen	
Cardiac	
Lung	
Vascular	
Performing POCUS takes too much time (all responses Strongly Agree, Agree, Disagree, Strongly Disagree)	
Lung	
Cardiac	
Abdomen	
Vascular	
I don’t use POCUS when I’m going to obtain a formal study (e.g. TTE) (all responses Strongly Agree, Agree, Disagree, Strongly Disagree)	
Cardiac	
Lung	
Abdomen	
Vascular	
I don’t use POCUS because my PE is sufficient to make the diagnosis (all responses Strongly Agree, Agree, Disagree, Strongly Disagree)	
Cardiac	
Lung	
Abdomen	
Vascular	
A barrier for me with traditional portable US is: (all responses Strongly Agree, Agree, Disagree, Strongly Disagree)	
Uploading images	
Carrying/transporting US	
Labelling images	
Access to a device	
A barrier for me with Butterfly is: (all responses Strongly Agree, Agree, Disagree, Strongly Disagree)	
Uploading images	
Carrying/transporting US	
Labeling images	
Access to a device	
I don’t feel comfortable using POCUS in front of the house staff (all responses Strongly Agree, Agree, Disagree, Strongly Disagree)	
When on service, how often do you scan? (Daily, Every 2-3 days, Once a week, Only with the right patient)	
What percentage of scans do you upload? (0-24%, 25-49%, 50-74%, 75-100%)	
What are your barriers to performing US when you are on service? (free text)	
What are your barriers to uploading the images? (free text)	
How can we help you perform more US and upload more images? (free text)	
Did you meet your learning goals? (free text)	
Reflecting on this program, what worked well and what didn’t work for you? (free text)	
Did you receive sufficient support from this program? (free text)	
Did you receive sufficient feedback on your skill development? (free text)	

## Abbreviations

POCUS: Point-of-Care Ultrasound
I-ScaN: Integrated Sonography Course at NYU
SHM: Society of Hospital Medicine
